# Sudden maternal hypoxemia during elective cesarean section in a woman with placenta previa

**DOI:** 10.1002/ccr3.1142

**Published:** 2017-09-02

**Authors:** Takeshi Umazume, Satoshi Hayasaka, Fumi Kato, Satoshi Ishikawa, Mamoru Morikawa, Hisanori Minakami

**Affiliations:** ^1^ Department of Obstetrics Hokkaido University Graduate School of Medicine Sapporo Japan; ^2^ Department of Anesthesia Hokkaido University Hospital Sapporo Japan; ^3^ Department of Diagnositic and Interventional Radiology Hokkaido University Hospital Sapporo Japan

**Keywords:** Amniotic fluid embolism, anaphylaxis, C1 esterase inhibitor, maternal mortality, pulmonary thromboembolism

## Abstract

There have been no reports regarding imaging‐documented bronchospasm in patients with amniotic fluid embolism (AFE). In a woman with scheduled cesarean section for placenta previa, transient bronchospasm and pulmonary hypertension were documented explaining a sudden drop in SpO_2_. Mild AFE was the most likely diagnosis in this patient.

## Introduction

Amniotic fluid embolism (AFE) remains one of the most devastating conditions in obstetric practice, with an incidence of approximately 1 in 40,000 deliveries and a reported mortality rate ranging from 20% to 60% 1. The clinical similarities of this condition to other types of acute critical maternal illness and the broad spectrum of disease severity hamper progress in our understanding of this syndrome. Sudden hypoxemia, hypotension, and coagulopathy with onset during labor or immediately after delivery are hallmarks for a diagnosis of amniotic fluid embolism 2. However, the mechanisms leading to hypoxemia in AFE are not well understood, although pulmonary hypertension at the initial phase of AFE has been documented in some cases of AFE 3, 4, 5, 6, 7, 8.

Risk factors for AFE include cesarean section with or without labor and placenta previa 9, 10, 11, 12, 13; for example, women with cesarean section without labor and those with placenta previa were reported to have 8.1‐fold and 10.5‐fold higher risks of AFE, respectively, compared to those without the respective characteristics in New South Wales, Australia 13. Here, we describe a woman that experienced dry cough with a subsequent gradual decrease in blood pressure prior to a sudden fall in PaO_2_ 45 min after childbirth with scheduled cesarean section for placenta previa at term. The patient gave informed consent for this presentation.

## Case Presentation

**Figure 1:**
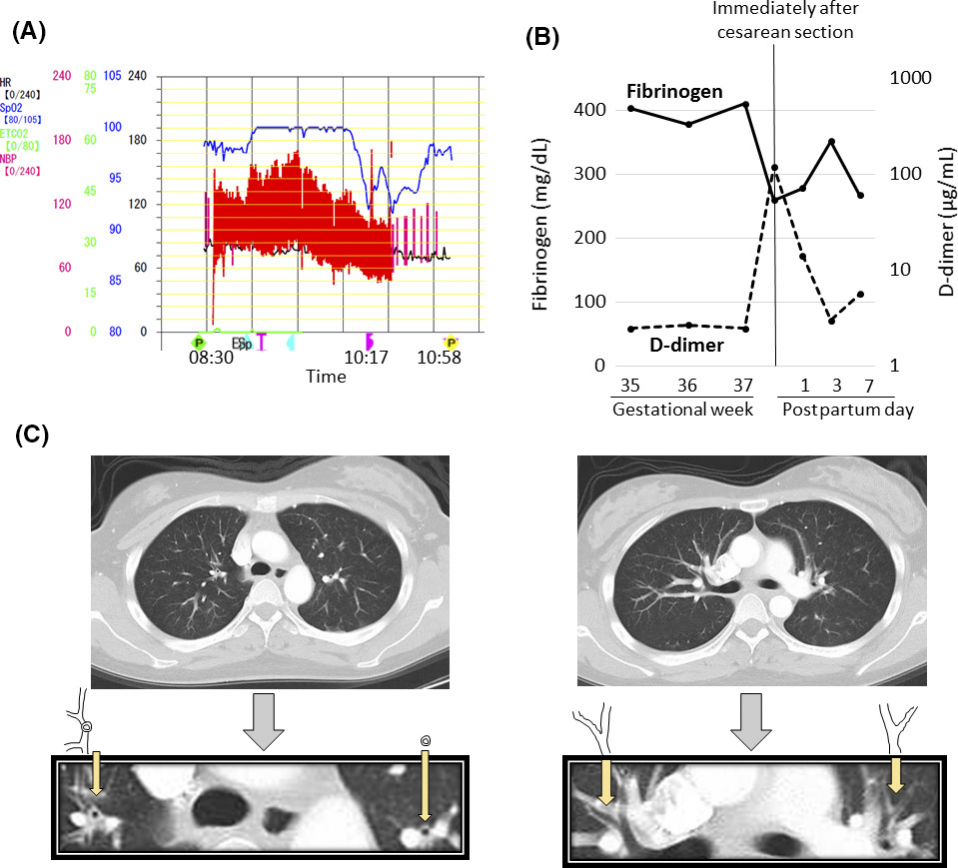
Findings in a woman with abrupt hypoxemia during cesarean section. (A) Changes in SpO_2_ (indicated in blue), blood pressure (in red), and heart rate (in black) around the hypoxemic episode. (B) Perinatal changes in blood fibrinogen and D‐dimer levels. (C) Computed tomography performed at 11:33 indicating thickened bronchus walls. Dry cough occurred at 10:00 and blood pressure 123/62 mmHg at 10:07 fell to 98/50 mmHg at 10:17. Hypoxemia occurred suddenly at 10:17, and normalization of SpO_2_ occurred at 10:58. PaO_2_ was 113, 225, and 68 mmHg, at 08:59, 09:50, and 10:20, respectively.

A 28‐year‐old nulliparous Japanese woman with prepregnancy body mass index of 23.6 kg/m^2^ underwent scheduled cesarean section for placenta previa at gestational week 37‐3/7. Her medical history was unremarkable, and she had never experienced an asthmatic attack. Her pregnancy was uneventful. In the morning of cesarean delivery, combined spinal and epidural analgesia was given, and cesarean section was started at 09:26 with latex‐free procedures. She gave birth to a healthy female infant weighing 2555 g with 1‐ and 5‐min Apgar scores of 7 and 9, respectively, at 09:32. Blood loss including amniotic fluid was 1430 mL until the time of abdominal wall closure, at which time a hypoxemic event evidenced by a sudden fall in SpO_2_ reading (from 100% to 92% under 6.0 L inhaled oxygen via a mask) occurred at 10:17 and lasted for 41 min until 10:58 (Fig. 1A). Dry cough occurred several times at 10:00 and a gradual decrease in blood pressure began at 10:07, reaching 98/50 mmHg at 10:17, preceding the event in this patient. PaO_2_ was actually low (68 mmHg at 10:20), and the results of blood tests at 10:45 were as follows: hemoglobin concentration, 9.4 g/dL; platelet count, 182 × 10^9^/L; fibrinogen, 259 mg/dL; antithrombin activity, 69%; high D‐dimer level, 119 *μ*g/mL (Fig. 1B); subnormal level of C1 esterase inactivator, 68%; and normal levels of complement components (C3, 94 mg/dL; C4, 31 mg/dL; and CH50, 45 U/mL). Drugs and fluid replacement given in this patient before and after the event are listed in Table 1. Transthoracic echocardiography performed during hypoxemia (at 10:45) suggested pulmonary hypertension with leftward deviation of interventricular septum (photographs of echocardiograms were not available). Chest computed tomography (CT) scan performed at 11:33 indicated thickened walls of bronchi and no thrombi, suggesting bronchospasm but not pulmonary thromboembolism (Fig. 1C). Neither coagulopathy, uterine atony, nor shock ensued. The postoperative clinical course was uneventful, and the patient and her daughter left hospital on postpartum day 8.

**Table 1:** Drugs and fluid replacement given in this patient during cesarean section

Until onset of hypoxemia (at 10:17) after entering the operating room (at 08:24)
20 mg of lidocaine at 08:51 into the epidural space
10 mg of bupivacaine (hyper) and 10 *μ*g of fentanyl at 08:55 into the subdural space
100 and 50 *μ*g of iv phenylephrine at 09:33 and 09:53, respectively
0.2 mg of iv methylergometrine at 09:33
10 mg of iv metoclopramide at 10:02
10 units of iv oxytocin and 10 units of oxytocin into uterine muscle at 09:33
1100 mL of iv bicarbonate Ringer's solution
500 mL of iv hydroxyethyl starch solution (Voluven^®^Fresenius Kabi Japan, Tokyo, Japan)
Until recovery from hypoxemia (at 10:58) after onset of hypoxemia (at 10:17)
100 mL of iv autologous blood starting at 10:27
350 mL of iv bicarbonate Ringer's solution

iv, intravenous.

## Discussion

This patient developed sudden hypoxemia at the time of abdominal wall closure preceded by dry coughing and a gradual decrease in blood pressure after uneventful elective cesarean section at term for placenta previa. Echocardiography during hypoxemia suggested pulmonary hypertension, and chest CT 32 min after recovery from hypoxemia indicated thickened bronchus walls suggesting bronchospasm. Thus, this patient may have suffered from hypoxemia caused by pulmonary hypertension and narrowing of the airways. It was speculated that narrowing of larger airway canals would have been seen if chest CT had been performed during hypoxemia.

Bronchospasm and pulmonary hypertension have a common pathology, that is, smooth muscle constriction of trachea/bronchioles and pulmonary arteries/arterioles, respectively 14. Recent case reports demonstrated pulmonary hypertension on echocardiography in patients with acute phase AFE 3, 4, 5, 6, 7, 8. The pathophysiology leading to AFE appears to involve an abnormal maternal response to fetal tissue exposure associated with breaches of the maternal–fetal physiological barrier during parturition 2. Bronchospasm and or laryngeal edema can occur in patients with anaphylaxis 15. As trigger(s) of smooth muscle constriction were not specified in this patient, differential diagnoses included mild AFE and mild anaphylaxis against undetermined substance(s) given during cesarean section. However, mild AFE was the most likely diagnosis based on circumstantial evidence, including scheduled cesarean section for placenta previa under latex‐free conditions (the usual procedure in our institution) and timing of the episode, that is, 45 min after childbirth; the former is a well‐known risk factor for AFE 9, 10, 11, 12, 13, and the latter is a well‐known danger period for the occurrence of AFE 2. In addition, this patient coughed preceding the event, and coughing is listed as a clinical sign of AFE 12.

Coagulopathy and hypofibrinogenemia are characteristic clinical features of typical AFE 2. The hypoxemia resolved spontaneously (at 10:58) without any specific treatment 41 min later, and coagulopathy was not seen, but the increase/decrease in D‐dimer/fibrinogen was marked in this patient. The lack of coagulopathy and transient hypoxemia was reasonable if this patient had mild AFE.

The decrease in blood pressure preceding hypoxemia was not explained by vascular smooth muscle constriction. Causes of hypotension in typical AFE patients are not well understood. It is unknown whether the decrease in blood pressure prior to hypoxemia is relevant to AFE, and it was possible that the decrease in blood pressure merely reflected blood loss in this patient. The hypotension of typical AFE can be considered as originating from left ventricular failure following pulmonary hypertension 11 and/or hypovolemia due to massive bleeding caused by coagulopathy. Our patient did not exhibit left ventricular dysfunction or coagulopathy.

To our knowledge, there have been no reports regarding imaging‐documented bronchospasm in AFE patients. Further studies are needed to determine whether bronchospasm in addition to pulmonary hypertension is a contributing factor leading to hypoxemia in AFE patients.

## Authorship

TU and HM: drafted the manuscript. TU, SH, FK, SI, and MM: treated the patient. All authors: participated in discussion about the patient and approved the final manuscript.

## Conflict of Interest

None declared.
